# What Is the Effect of Cultural Greenway Projects in High-Density Urban Municipalities? Assessing the Public Living Desire near the Cultural Greenway in Central Beijing

**DOI:** 10.3390/ijerph19042147

**Published:** 2022-02-14

**Authors:** Haiyun Xu, Fan Fu, Meng Miao

**Affiliations:** 1 School of Architecture and Urban Planning, Beijing University of Civil Engineering and Architecture, Beijing 100044, China; xuhaiyun@bucea.edu.cn; 2 School of Finance, Renmin University of China, Beijing 100086, China; miaomeng@ruc.edu.cn

**Keywords:** cultural greenway, urban green space, public living desire, housing condition, housing market, urban planning

## Abstract

Cultural greenway projects (CGPs) are widely regarded as an urban planning approach which connects open green spaces and sites of sociocultural value to provide access to living, working and recreational spaces and enhance local social well-being. This paper examines the impact of such CGPs on public living desire before and after a given project is completed through analyzing housing prices in the surrounding area. We deployed a hedonic pricing model (HPM) and differences in differences (DID) model to analyze and record any changes in housing market trends that may have been caused by such a cultural greenway project. Via analysis of single-family home sale transactions in central Beijing from 2013 to 2017, we found substantial evidence that proximity to a cultural greenway project is positively linked with rising property prices. Once complete, CGPs were similarly associated with positive increases per HPM and DID modeling. Our results revealed that the distance to greenway contributed significantly positive impact on the housing market after the cultural greenway project completed. Moreover, our result indicated that once a CGP was open to the public, it increased the price of properties within 1 km by 13.3%. Seller and buyer expectations of the development of local, green public infrastructure also began to factor into housing prices prior to the greenway opening to the public. Post-completion, the positive trend in property pricing due to local CGPs indicates that the public still have an increasing desire to live near the greenway. These results will help policymakers better understand how cultural greenways affect neighborhoods in high-density urban contexts, and will support the development of urban greenway policies for cities in China that reap the maximum economic benefit.

## 1. Introduction

As linear corridors of green space, greenways have the capability to connect communities and protect important cultural, ecological and recreational resources within a region. Previous research has described the diverse benefits that these greenway projects have on both the ecological and cultural values of their surrounding areas [1,2,3]. Among them, cultural greenway projects (CGPs) connect open green spaces and sites of sociocultural value to provide access to living, working and recreational spaces and enhance local social well-being to provide a link between neighborhoods, and supply increased opportunities for recreation and socio-environmental revitalization [1,3,4,5]. Since the 1990s, greenway planning had already attracted increased attention from both scholars and regional administrators as a potential avenue to foster connections between local cultural and natural resources—resources which could also contribute to local development through the provision of multiple functions such as recreation, biodiversity conservation, heritage, education, and so on. Greenway planning projects have thus been promoted across the world for decades now by countless urban administrators [6,7,8,9,10]. For high-density urban municipalities, greenways are commonly regarded as just as valuable a green resource as other green open spaces; indeed, both equally serve to improve quality of life within neighborhoods, which are often crowded due to land constraints and high population. Moreover, due to the linear nature and length of greenways, they often span multiple political and jurisdictional boundaries, and affect a more complex array of stakeholders than other types of green space project [6]. However, since CGPs in an urban context rely on the integration of cultural, historical, and civilian values—mostly in the form of space and infrastructure—it is valuable to understand and quantify the extent to which cultural greenway projects influence neighborhood estate prices in high-density urban municipalities like central Beijing.

Current literature on greenway project contributions mainly focus on their ecological benefits, such as climate regulation, carbon sequestration, oxygen production, and biodiversity conservation [7,8,9,10]. Even though the social aspects of greenways has attracted increasing research attention in decades in both China and western world [11,12,13,14], few existing studies quantify and evaluate the social-economic benefit of CGPs, nor do they record their contribution in landscape performance series (LPS) analyses, various ecosystem service value assessments, cost-benefit analyses, travel cost analysis (TCM), the contingent valuation method (CVM), nor the cross-sectional hedonic price method using past sales transactions [15,16,17,18]. What few studies there are do show that greenways are regarded as amenities by residents. They are willing to pay extra to travel for recreation to such greenways, and are willing to pay more for properties near greenways to live around. This public living desire may in fact lead to the price fluctuations we discovered in the local housing market near CGPs. Prior studies have also deployed hedonic price studies to show a positive association between natural amenities (including greenways) and neighborhood property values [19,20,21]. These studies mainly focus on examining the influence of natural amenities in general as opposed to greenways alone, which means the characteristics of cultural greenways in high-density urban contexts are rarely considered.

Interestingly, there are almost no studies that examine the influence of CGPs on public living desire in surrounding areas, particularly in the context of increases to greenway development and planning in China. These recent increases are perhaps best hallmarked by the construction of the very first greenway network project in China, the Pearl River Delta Greenway Network. Built in 2009, the grand opening of this location was regarded by some as a hallmark event, notably attracting considerable policy attention across China [22]. Following this endeavor, the Ministry of Housing and Urban-Rural Development established a greenway corridor and network initiative in 2016 that was intended to accelerate the construction of greenway projects along a variety of spatial locations throughout the country [23,24]. Such changes even sparked a “boom” in the various greenways facilitated by those central or local governments with more relaxed fiscal budgets [23]. All that being true, current studies on the contributions of various natural amenities, including green spaces and greenways, are mainly based on European and American contexts [25]. Indeed, there have been few studies undertaken in China, with the only existing study being focused on public green spaces [26,27]. Furthermore, no study of this kind, to the best of our knowledge, has explored the economic influences and public living desire changes on neighboring residential property values caused by cultural greenway completion and development in the context of China. We believe this absence to be surprising in consideration of the so-called boom in greenway planning and research projects over the last few years.

Our research thus examines the influence of cultural greenway projects through assessing public living desire based on neighboring residential property values in high-density urban contexts. This study focuses in particular on the Huanerhuan Greenway, which is located around the old town of Beijing, and was originally based on the layout of historical city walls and moats. This greenway is located in the capital core area of Beijing, which happens to be one of the most built-up, highly-dense urban areas in China. As such, this greenway project was planned to spur urban renewal amid the central historical district of Beijing, and was completed in 2015. We deployed both a hedonic pricing model (HPM) and a differences in differences (DID) model to analyze associated trend changes and impacts in nearby housing prices via spatial regressions completed before and after the completion of the Huanerhuan Greenway project. The HPM is a pricing model of value, the benefits of which consider both internal and external factors [28,29]. It is a model often applied to evaluate the economic values known for non-market environmental amenities and services—specifically those that directly affect market prices. In essence, it values the price that people are willing to pay to experience a change in the environmental characteristics that surround them, such as better recreation functions and air quality [30,31]. On the other hand, the DID model is one of the most frequently used methods during impact evaluation studies [32]. This method instead calculates the effect of a treatment on an outcome via the combination of before–after and treatment–control group comparisons [32]. Such a model has an intuitive appeal and has been widely used in public policy, health research and management fields, for instance, to estimate the effects of public and health care policies [33,34]. As such, we used HPM to analyze the before and after impacts of a cultural greenway project on the single family housing market, and the DID model to examine the changes of such impacts. Our study raises the following research questions:

- What effects does the opening of this cultural greenway project have on the local housing market?
- What levels and trends of nearby pre- and post-housing prices are related to the opening of this cultural greenway project’s impact?

We begin by drawing on the literature to explain the need to assess the economic contributions made by greenway projects, and their influence on local public living desire. The rest of the paper is organized as follows: Section 2 describes the materials and methods and justifies the differences between the HPM and DID models. Section 3 describes the main findings of the paper, while Section 4 discusses our understanding of the results and comparisons found with other cities, as well as the limitations. These explorations are followed by the conclusion and perspectives in Section 5.

Our study not only provides a better understanding of the influence that cultural greenway projects have on public living desire—through analysis of housing prices within a certain proximity to central Beijing—but also offers a new perspective for potential investment strategies by city policymakers during urban green infrastructure planning initiatives.

## 2. Materials and Methods

### 2.1. Study Area

**Figure 1:**
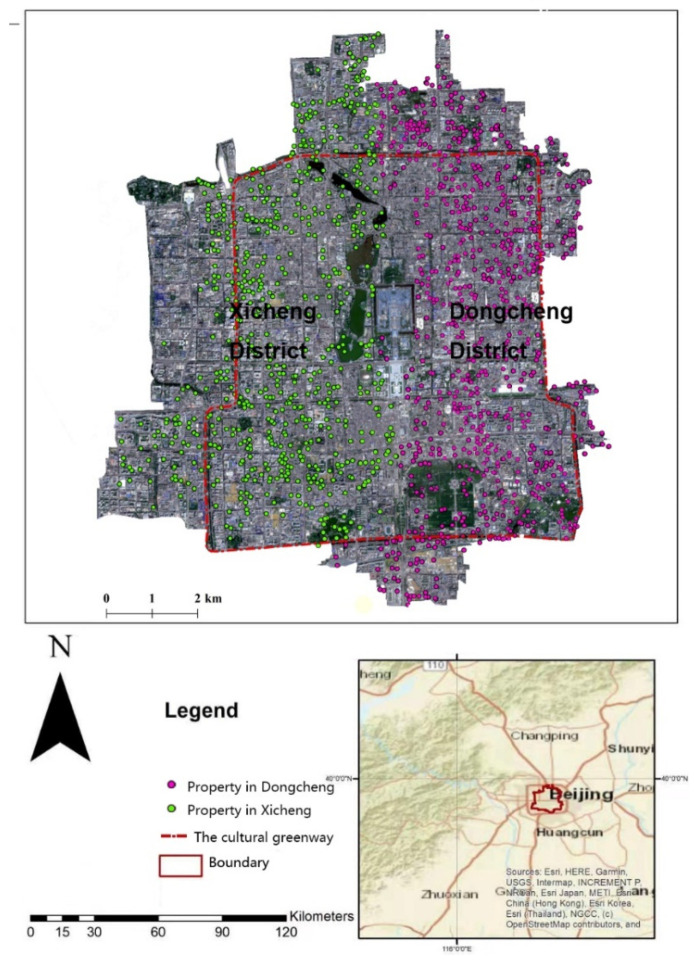
Location map of the Huanerhuan cultural greenway project that displays the distribution of recorded property sites in nearby housing markets in the capital core area of Beijing.

Our study area focused on the “capital core area” of Beijing, which itself is the capital city of China, and the most densely populated city. Because Beijing is a historical city, the central core of the city—including the Dongcheng and Xicheng districts of Beijing that span an area of 92.5 km^2^—is endowed with the full function of the country’s political, cultural, and international exchange [7,33] (Figure 1). The area also serves as a key conservation area for Chinese historical and cultural heritage, and offers a window through which to assess the greater urban area in and around Beijing. Its population density is among the highest in the world, at 22,849 persons per km^2^. Likewise, housing prices in Beijing are among the highest countrywide, and vary greatly across the city. There exists a huge gap between the housing prices in central Beijing and surrounding sub-areas, and between normal, non-adjacent communities and neighborhoods with good primary schools—each of which are mainly located in Beijing-center. According to the “Nearby Enrollment Policy of the Compulsory Education in Beijing”, each city block has a corresponding primary school, referred to as a school district during the school year. Due to this policy, the housing prices in the capital core area of Beijing have increased more rapidly than most over the last decade. Indeed, average property prices were about 32,000 CNY/m^2^ in 2010, growing to 121,000 CNY/m^2^ in 2021.

The Huanerhuan Cultural Greenway was formally completed by 2015 [24]. This greenway project was planned along the Second Ring Road in Beijing, and was based on the layout of ancient city walls and moats of historical Beijing. Project planning commenced in 2012, with project activity starting in 2014. Overall it took three years of planning and construction works to transform the local brownfield, the abandoned riverside, demolish any illegal camps or obstructions against the city wall, convert the abandoned riverside, and improve the urban lost spaces into a 34.5 km cultural greenway with recreational bike and pedestrian paths [34,35]. The greenway linked more than 20 park green spaces and cultural relics, such as the Temple of Heaven, the Temple of Earth, and the Lama Temple. This greenway similarly integrates the second ring road, moat road green space, riverside green space, and large swaths of residential area containing parks, schools, shopping areas, and transit stops. As such, it has now become the most important landmark greenway in terms of showcasing the capital’s pride in its historical and cultural features, and economic and social development. Incredibly, this CGP crossed more than 40 communities in the capital core area of Beijing, making it the green space system project with the widest area, thereby affecting those citizens in high-density areas of central Beijing to a greater degree than those in neighborhoods far away [34,35].

### 2.2. Data Collection

Our study included both quantitative and qualitative methods. In our pilot study, we collected related literature and documents on the Huanerhuan cultural greenway from the Beijing planning department and social media. Our research team also conducted random face-to-face interviews and field observations along with the study area in order to understand the site’s transformation before the construction of the cultural greenway project and local public preferences. All interview contents and observation notes were recorded as soon as possible. In the formal study, we used single-family home sales transactions recorded from January 2013 to December 2017 in the capital core area of Beijing, including sale records from the Dongcheng and Xicheng districts. Here, market price is the preferred measure of value, because it directly reflects individuals’ fiscal allocations, themselves likely formed by competing home buyer valuations for houses on the market at that time [36]. We used a requests library to crawl the online housing transaction information from Lianjia.com, then analyzed the page through Beautiful Soup to gather house sales transaction histories and property characteristics data, including house price, location, house type and so on. The original sales transaction data included 57,402 single-family property transaction records. Property characteristics data included information such as the scale, year of build, number of bedrooms, how many stories and elevators, as well as binary variables indicating whether the property has a school near the estate in accordance with local school district policy. Based on the longitude and latitude (WGS1984) of the sourced property transaction data, we transformed the above data into point data, and entered this into ArcGIS (Esri, Redlands, CA, USA). We also used the land cover dataset from the Finer Resolution Observation and Monitoring of Global Land Cover (FROM-GLC10) for this study, interpreted from 10 m resolution satellite imagery collected via Sentinel-2 in 2017 [37].

Sentinel-2 (Esri, Redlands, CA, USA) was launched in 2015, and conducts global observation tasks via high-resolution, multispectral imagery. This satellite delivered 13 spectral band satellite observation products, including the 10 m resolution optical and near-infrared bands relevant to our study. A supervised machine learning method was applied to complete the classification task. Thus, both training datasets and validation datasets were collected based on Landsat 8 imagery taken from observations made in 2014 and 2015. Based on this data, we mapped the spatial pattern of Huanerhuan Cultural Greenway. The location of the cultural greenway project and the distribution of property record sites are shown in Figure 1 below.

Based on this map, we calculated the distances between these properties and target amenities such as greenways or substations using ArcGIS 10, and recorded outcome distances. To record the characteristics of this greenway project, we also collected the distance between each property data point and the nearest cultural heritage site. Even though the quality of primary schools in our study area was high in the context of Beijing, there were still considerable gaps between highly sought-after elementary schools and public schools. Thus, we also collected the school district information associated with each property based on their location in the ArcGIS 10. Based on primary school location and level, we ranked properties in multiple school districts into three levels: school estate at the city level plus key elementary school (record score = 2); school estate at the district level plus key elementary school (record score = 1), and school estate near public school (record score = 0). In general, we collected the variables across four dimensions: (1) dependent variable: the price of each property; (2) nature-environment variable: the distance to greenway, which is the key variable in our study; (3) location variables: distance between property and subway station, shopping center or heritage sites and school estate level; and (4) structural characteristics: scale of each house, number of bedrooms, how many stories, year of construction, and elevator status. The summary statistics for these variables are presented in Table 1 below.

### 2.3. Data Analysis

In this study, two different methods were applied. First, we use the hedonic price model (HPM) to examine the before and after impacts of a given cultural greenway project on the nearby housing market to assess the public living desire change from development to completion. Then, we applied the difference in differences (DID) model to examine the influence level and impact trends of the cultural greenway project on property prices in the vicinity.

#### 2.3.1. Hedonic Pricing Model

HPM identifies the impact factors and characteristics that affect an item’s price in the market [36]. This model is commonly used in the housing market, since real estate prices are determined by the characteristics of the property itself as well as the neighborhood or environment within which it exists. The HPM captures consumers’ willingness and desire to pay for what they perceive are environmental differences that add or detract from the intrinsic value of a property. It is widely used to assess the living desire of people [36]. Thus, we used HPM to explore the influence of the completed cultural greenway project to the public living desire based on the housing market in the high-density area. The hedonic framework in this study is as follows:

(1) $$P=f\left(\underset{s,}{\to }\underset{n,}{\to }\underset{e,}{\to }\underset{T}{\to }\right)$$

where the price of a property (P) will thus be affected by the structural characteristics of the house itself (scale, bedrooms in the house, etc.)$\;\underset{s}{\to }=(s1,\;s2,\;s3\ldots$,) characteristics of the location (school estate, subway, etc.) $\underset{n}{\to }=\left(n1,\;n2,\;n3\ldots \right)$, and environmental characteristics (greenway project, etc.) $\underset{e}{\to }=\left(e1,\;e2,\;e3\ldots \right)$ (see Table 1). We also considered the time variables for each transaction, i.e., the year of sale, to account for yearly differences $\underset{T}{\to }=\left(T2013,\;T2014\ldots \right)$ in the housing market. The partial derivative of this function represents the implicit price for each characteristic or attribute.

#### 2.3.2. Difference in Differences (DID) Model

The DID model is typically used to estimate the effect of a specific intervention or treatment (such as a large-scale program implementation) by comparing the changes in outcomes over time between a population that is enrolled in a program (the intervention group) and a population that is not (the control group) [38]. The DID model is shown as follows:

Y= β0 + β1 × [Time] + β2 × [Intervention] + β3 × [Time × Intervention] + β4 × [Covariates] + ε(2)

where the treatment effect on the treated (causal effect in the exposed) (Y) will thus be affected by the baseline average (β0), the time trend control group (β1), the differences between two groups pre-intervention (β3), and the change differences over time (β4).

In our study, we regarded the construction of this cultural greenway as the specific intervention, and we used the difference in differences (DID) model to compare trend changes and other impacts on housing prices in the vicinity of the cultural greenway project, before and after it was completed, with those in neighborhoods outside of our target proximal area, i.e., where the greenway did not intersect.

We then used the DID model to assess the impact of the cultural greenway project on neighboring housing prices. The variables for this DID model were based on the HPM, and accounted for the structural and locational characteristics of properties in our dataset. It also clarifies the causal direction to capture differentials in levels and trends of pre- and post-housing prices related with the cultural greenway project’s completion.

## 3. Results

### 3.1. Hedonic Price Model

The resultant before and after models present almost identical coefficients for the structural attributes, and comparable coefficients for the location attributes (Table 2). In terms of the structural attributes, housing scale, number of bedrooms, property age, and elevator status are positively and significantly associated with property prices. However, apartment floors are negatively associated with property sale prices. Among all listed structural attributes, elevator status was revealed to be the strongest relevant variable in terms of public living desire and impacted property price. Properties listed with elevator access are valued 22% and 26% above normal before and after the CGP is complete, respectively. As we expected, location attributes also showed significance per school estate status and distance to a nearby subway station. As regards the listed location characteristic variables, those within school estate districts are the most relevant in terms of price. Apartments with good quality primary schools nearby have an increased property value of 106% and 155% per level, before and after completion, even when controlling for other factors. A property nearby a subway station also indicated significant increases to property sale value. Indeed, being located a single kilometer closer to a subway satiation increases the value of a given property by 5% and 10%, before and after CGP completion when controlling for other factors. However, this result also indicates that prior to cultural greenway project completion, local cultural heritage sites and a shopping center had no significant impact on property value, while being located a mile closer to a heritage site does significantly positively impact property values by 8% in the completed (after) CGP model, and a shopping center still has no significant impact.

Moreover, our focus variable for the study—distance to a greenway—indicates that there are pricing benefits to being near the greenway both before and after the cultural greenway project is complete. For example, prior to completion, a property located 1 km closer to the abandoned riverside, the lost spaces, and a brownfield site, as well as the lot spaces along the second circle road faced a 4% decrease in sales price when compared to similar properties outside of our target area. At the time that the cultural greenway project was underway, from 2015 to 2017, the same property experienced a 5% increase in sales price when compared to properties further away from the greenway development. Our HPM result thus indicates the significant impact that distance has on house pricing once the cultural greenway project is complete.

### 3.2. Difference in Differences Model

The DID model revealed coefficients that are similar to the coefficients observed by the hedonic price model presented above. The control variables in the DID model explain a higher percentage of variance than the HPM model. The key variables of the DID are the pre-impact price level, post-impact price level, pre-impact price trend, and post-impact price trend, all of which capture the pricing impacts before and after the completion of the CGP.

According to Table 3, the coefficient of the pre-impact level shows the following: before the cultural greenway project was complete (2013–2015), the average price level for properties within a 1 km radius of the cultural greenway project (still empty or lost spaces, abandoned riverside, and brownfields) are 3.2% cheaper than properties outside the 1 km radius. Additionally, the pre-CGP impact trend increased by 1.4% annually, likely as a result of the greenway nearing completion. This pre-impact trend result revealed that even before the greenway project was complete, buyer and seller willingness to live near the greenway tends to increase as the promise of public access comes closer. Such considerations then factor into property pricing in the local housing market.

On the other hand, once the cultural greenway was entirely complete, the average property price for homes within a 1 km radius of the cultural greenway grew 13.3% more than properties outside that target radius. The post-CGP impact trend similarly displays an increase in pricing premiums by 1.3% annually, and showed that, once complete, buyers and sellers continued to display increased willingness to live near the greenway, and lead to positive trends within the nearby housing market.

In sum, the use of the DID model resulted in indicators that the impact location of housing prices along a greenway space are defined within (and outside of) one kilometer.

## 4. Discussion

This study adds to an ongoing effort to compare the public living desire change through assessing housing market influences in high-density urban areas such as Beijing, before and after a cultural greenway (CGP) project has been completed.

Based on the interviews, we noted the residents’ strong preferences for local green space. For example, a participant living near Lianhuahe greenway, which is the southwest part of Huanerhuan cultural greenway, stated that “*this place used to be a stinky ditch and no one was here, while the new greenway becomes fishing places, and a flower viewing spot, and walking along the greenway for thousands of steps is the important way of exercising in our daily life now*”. This indicates that nearby residents preferred environmental, aesthetic and recreational value and regarded the greenway as scarce green space resources in the central old town of Beijing (i.e., not only for its function as a passageway). These everyday use and recreational value are in line with existing studies, namely, these preferences could be linked to the cultural and social functions provided by urban green spaces, which might contribute a healthy living environment for outdoor recreational and natural education opportunities and harmonious people–environment relationship [39]. Moreover, we realized that public preferences with the high-frequency daily use for nearby green space would facilitate engagement in the community, promote a local sense of belonging and social relations, conferring social and cultural connotations on green spaces, based on previous studies [40,41]. As a consequence, we believe that these preferences enhance people’s desire and willingness to pay extra to live around the green space and affect local property decision making.

Such effects have been found in some other cities. For example, Hong Kong residents are willing to pay extra 9.9 USD per month to live properties with green spaces for family recreation and exercise [42]. Furthermore, the residents’ positive preferences toward environmental and recreational amenities provided by green spaces leads to a positive effect (1% rise) on the prices of residential buildings in Prague [43]. Given lessons from other cities, we assumed that public preferences on green spaces will also positively affect their living desire on the properties near the CGP as a scarce green space resource in central Beijing. Then, we performed two models to verify the effect made by the cultural greenway on public living desire.

By employing both the HPM and the DID models, this study was able to analyze the general impact and the extent of the impacts and trends for the public living desire on the properties in the capital core area of Beijing located nearby the cultural greenway. Even though the HPM model did reveal the general influences of a CGP on the nearby housing market (before and CGP completion), it cannot explain why the cultural greenway was the direct reason for such changes. Instead, it serves to estimate the impacts of a cultural greenway project as a specific green infrastructural intervention. Previous studies have addressed the potential for the DID model to reveal impact associations and changes due to a cultural greenway project being built [44,45]. At present, the model could reveal the trend of such changes, and even strengthen the statistical robustness of our results against the threat of external events [44]. We thus performed the DID model as a supplementary model to directly estimate the impact change of a cultural greenway project, and to explain change trends. It also partially verifies causality via a comparison of both the influence and control areas. The two models together present similar coefficients for pre-complete and post-complete variables, which confirm changes to how cultural greenway project completion influences surrounding neighborhoods. In general, our study indicated that the construction and completion of a new cultural greenway has a significantly positive affect on the nearby housing market. This result is similar to pervious gentrification studies, in which scholars have addressed how urban greening initiatives and green space restoration can raise property values and lead to local gentrification [46,47].

Besides, we revealed those variables which significantly affect property prices in our study. Our results indicate that the key variable of the study—the distance to a greenway—contributed a significantly positive impact on the public living desire through assessing the housing market after the new greenway project was completed. Before completion of the CGP, however, for every kilometer that a property gets closer to the riverside and the lost spaces along the second circle road, the sale price of the property becomes 4% cheaper than other properties further away. Once the cultural greenway project was completed between 2015 and 2017, properties then became 5% more expensive for each kilometer moving toward the cultural greenway project. The results we found for the general impact that proximity to a greenway has on the housing market is similar to the green space’ impact in previous cases in Europe and the US [25,48,49,50]. Moreover, we found that building scale, the number of floors, year of construction, elevator situation, whether the property is near a school estate, and the distance to a subway station were significant variables affecting property prices in our study. Similar results have been found in previous HPM studies on urban green spaces and greenways [27], adding to the importance of our results.

Our findings also indicate that in the capital core area of Beijing, school estate locations possess the most relevant effect variable in terms of price, followed by elevator status. These results encompass our entire study period from 2013 to 2017. Since Beijing also has the most concentrated area of educational resources across all of China, many previous studies have addressed school estate premiums as being one of the strongest influences on local property prices [51,52]. Besides, the high relevance of elevator status on property price may be explained by our study on the old, historical area of Beijing, which houses a high number of aging, residential communities. Studies completed on the topic of these older residential communities and the best practices for renovation see elevators as the one of the most important factors enhancing local resident wellbeing [53,54] in an urban context. Nonetheless, the results did show a difference in pricing for houses on the market prior to project completion, whereby the distance to heritage sites rose to positively and significantly impact property price rise after the cultural green way project was complete. These findings are similar to those of previous heritage conservation studies, which address property price near conservation areas, and estimate increases once the heritage conservation project is open to the public [55]. Furthermore, these results could also be explained by the characteristics of a given cultural greenway project. As cultural greenways connect cultural resources with green open spaces and human passages, these heritage sites are always planned as critical scenic locations that gain sufficient investment to enhance the nearby environment. As such, and in accordance with those other studies, land use for recreational and cultural functions may also affect nearby property prices [45].

At this point, our results indicated a positive impact level and positive impact trend for the contributions of cultural greenway projects on neighboring public living desire. Prior to building the CGP, these areas consisted of abandoned riverside, lost spaces, illegal constructions, and brownfield sites along Beijing’s second circle road. For example, a property located in any neighborhood within a 1 km radius of the planned project area becomes 3.2% cheaper than properties outside of the 1 km radius, before the cultural greenway project is complete. After the cultural greenway project is complete, average property prices within that one-kilometer radius (of the cultural greenway) became 13.3% more expensive in sale price compared to properties outside that area. This positive impact trend in our result was similar to previous studies on urban parks, and their effect on the housing market in Beijing. (between 0.5–14.1%) [26,56]. Similar impact trends have been found for urban green spaces in previous reviews, which demonstrate urban green spaces as having a 5% to 20% premium on neighboring property values [25]. As regards the impact trend associated with the cultural greenway’s construction, our result did indicate that pre-impact trends increased by 1.4% annually as the opening of the cultural greenway approached. This result indicates that public living desire and preferences for the greenway tend to increase near project completion, particularly as the redevelopment of physical surroundings becomes perceptible. These findings show that citizen expectations about the greenway being publicly accessible as well as an open green space translated into the housing market even before the project was completed and opened.

This positive trend result is similar to previous DID case studies focused on transforming abandoned railway land and brownfields into greenway projects or other recreation-based urban projects [44,45]. They also address the similarly negative impact that abandoned land has on surrounding property prices before these new urban planning projects are complete, as well as highlighting the positive impact made by these projects as well. However, they also estimate that there will be a decrease of that annual impact trend after the project is complete if residents feel the space does not meet their initial expectations—in which case these greenway and park projects ultimately fail to satisfy local residents [44]. Our result differs from prior cases; indeed, we see clearly the annual positive impact trend post-project completion rise by 1.3%. From our study, people maintain an increased desire to live within the vicinity, knowing the cultural greenway will satisfy their recreational needs. It is of course important to note that the scarcity of green space projects in Beijing’s historical district—in consideration of its status as a high-density urban context—may better explain this outcome. Whatever the case, enough studies have proven the critical importance of green spaces to enhance citizen quality of life in a high-density urban context [57]. In this context, cultural greenway projects indeed provide open, green spaces to improve local wellbeing [58,59].

However, some limitations should be noted. Firstly, our study mainly focused on the extent of the impacts and trends made by the CGP. The spatial patterns of public living desire and the changes affected by the CGP during planning, construction and public service after opening remain to be explored. More spatio-temporal elements and visualization elements are thus required for future studies that explore these variations.

Although the opening of a new cultural greenway project enhances local resident well-being and public living desire, increased housing prices can be a driver of community gentrification [46]. This gentrification could have a negative impact on residents, particularly on their sense of belonging and green space use [46]. The implications of gentrification, as interesting and important a topic as it may be, is beyond the scope of this paper. Future studies are needed to explore the long-term impacts of a CGP on the surrounding neighborhood in order to verify the possible consequences of community gentrification.

This study may, however, be limited in terms of generalizability, as it relates to the features of our selected study area. Indeed, Beijing is China’s capital city, and is the most population-dense city in the country, with our study largely focused on old town areas. At the same time, it is still not clear whether such an impact exists for different factors, like local land use policies (i.e., multifamily, commercial, or industrial), or city scale and context (i.e., larger or smaller, and older or newer). These unknown considerations may in fact limit our ability to generalize the results to other cities. Thus, explorations per the influence and contribution of greenway infrastructure in different urban and rural contexts should be the subject of future studies.

## 5. Conclusions

Our study assessed the effects of a new cultural greenway project (CGP) on public living desire through direct analysis of the local housing market. Despite the fact that there have been many scholars who report a positive relationship between green spaces and neighboring residential property values, we believe our paper makes significant contributions along the following three directions: firstly, previous papers mainly focus on general green spaces, while our study focuses on CGPs. Due to the features known to CGPs (e.g., social-cultural values, crossing communities), there is no guarantee that a greenway would affect the public living desire in the same way as a non-cultural green space, which indicates the necessity of a study that exclusively focuses on the features and impacts of a greenway project. Apart from a focus on assessing the ecological benefit of greenways in current studies, our paper quantifies the socio-economic contribution of CGPs relating to public living desire. Moreover, even though China has promoted a “boom” in the popularity of greenway planning projects for more than 10 years, few studies examine the economic contributions brought about by these greenways and how they impact public living desires within a high-density urban neighborhood context. This gap calls for further systemic and quantitative assessment.

Thus, in this article, we used a CGP case in China—the Huanerhuan cultural greenway in the capital core region of Beijing—to assess the contribution made by the project on public living desire in a high-density urban context.. Our study provides compelling evidence via estimations surrounding the influence of CGPs on public living desire through an analysis of urban property values near it. More specifically, we isolate the causal impacts of cultural greenway projects at the intersection of neighborhood heterogeneity. Our results show that cultural greenway projects have a positive impact on surrounding housing prices, a finding that is reflected in both the HPM and the DID models. In both models, it was not only the post-construction period but also the pre-construction period in which the cultural greenway projects showed a positive impact on that neighborhood’s property market. These results revealed the contribution of cultural greenway projects to a high-density urban context during the greenway planning boom currently underway in China. Urban decision makers could advocate the feasibility of these types of revitalizations as major contributors to urban renewal in aging areas of an urban city.

In closing, the last decade has seen an increase in greenway projects being promoted across the whole of China, usually by administrators from central and local governments. These projects may bring about a shift in otherwise abandoned riverside green spaces, ‘lost’ spaces, brownfields, old illegal constructions, and other urban spaces with social or ecological loss. This improvement would be due to the greenway’s provision of multiple cultural and ecological functions towards improved urban quality of life. Finally, this study revealed a local citizen preference for CGPs in high-density areas, as well as determined the variables which contribute to, and provide a positive impact on, local neighborhoods in the area.

More research is required to explore the long-term impacts of a new cultural greenway project. This positive effect on price increases within the local, burgeoning housing market may lead to gentrification in the future. The following aspects are thus proposed for future research: (1) to analyze the effects of local greenway projects for different resident groups, such as apartment owners and tenants, or local residents and non-local residents; (2) to directly assess resident perception on the new greenway project such as their degree of satisfaction, sense of belonging and sense of safety; and (3) to explore public use cases and activities related to the new greenway project based on long term multi-source social activity data. In terms of generalizability limitations, the comparative studies on different impacts made by a new greenway project in various cities with different urban factors (e.g., land-use policies, city scale and context) must also be explored in the future.

## Figures and Tables

**Table 1:** Descriptive statistics.

Variable	Mean	SD	Min	Max
Average sale prices (10,000 RMB)	503.985	900.336	1	19,000
Nature Environment				
Distance to greenway (km)	2.741	35.625	0.003	13.678
Location				
Distance to subway station (km)	2.108	34.113	0.031	13.642
Distance to heritage sites (km)	1.841	34.106	7.922	13.499
Distance to shopping center (km)	1.862	33.170	0.027	13.069
School estate level (0 = normal school, 1 = district-level key elementary school, 2 = city-level key elementary school)	0.108	0.310	0	2
Structural characteristics				
Scale of house (m^2^)	74.482	45.649	6.900	628.721
Number of bedrooms	1.942	0.769	0	9
Floor	12.415	6.887	1	31
Construction Year (AD1600–AD2017)	1994.349	10.217	1960	2016
Elevator status (yes = 1, no = 0)	0.578	0.493	0	1
Binary:1 = sale at year 2013	0.078	0.267	0	1
Binary:1 = sale at year 2014	0.106	0.307	0	1
Binary:1 = sale at year 2015	0.262	0.439	0	1
Binary:1 = sale at year 2016	0.368	0.482	0	1
Binary:1 = sale at year 2017	0.185	0.388	0	1

**Table 2:** Pre-completed and post-completed hedonic price model results.

Variables	Pre-Completed (2013–2015)		Post-Completed (2016–2017)	
	Coefficient	Robust Std. Err	Coefficient	Robust Std. Err
Distance to greenway	0.042 ***	(0.000)	−0.053 ***	(0.000)
Distance to subway station	−0.053 ***	(0.001)	−0.102 ***	(0.001)
Distance to heritage site	−0.002	(0.001)	−0.084 ***	(0.001)
Distance to shopping mall	−0.028	(0.018)	−0.031	(0.019)
School estate	1.061 ***	(0.294)	1.550 ***	(0.385)
Scales	0.053 ***	(0.004)	0.067 ***	(0.005)
Numbers of bedrooms	0.032 *	(0.018)	0.097 ***	(0.023)
Floors	−0.078 ***	(0.023)	−0.087 ***	(0.028)
Construction year	0.007 ***	(0.001)	0.007 ***	(0.001)
Elevator or not	0.220 ***	(0.031)	0.260 ***	(0.039)
Observations	20615		28222	
R-squared	0.682		0.660	

* means *p* < 0.1 and *** means *p* < 0.01.

**Table 3:** Differences in differences (DID) regression results.

Variable	Coefficient	Robust Std. Err
Impact_pre-level (2013–2015)	−0.032	(0.044)
Impact_post-level (2016–2017)	0.133 ***	(0.053)
Impact_pre_trend (2013–2015)	0.014	(0.038)
Impact_post_trend (2016–2017)	0.013	(0.036)
Distance to subway	−0.050 ***	(0.010)
Distance to heritage	−0.050 ***	(0.010)
Distance to shopping center	−0.028	(0.018)
School estate	1.342 ***	(2.585)
Scale	0.062 ***	(0.004)
Numbers of bedrooms	0.013 ***	(0.002)
Floors	−0.077 ***	(0.020)
Construction years	0.003 ***	(0.001)
Elevator or not	0.038 ***	(0.027)

*** means *p* < 0.01.
